# Mixed subtype of congenital mesoblastic nephroma with poor evolution: a case report and literature review*

**DOI:** 10.1590/0100-3984.2013.1613

**Published:** 2015

**Authors:** Sydney Correia Leão, Diego Marques Fernandes, Bruno Garcia Dias, Wlisses Ramon Oliveira, Simone Maria de Oliveira, Margareth Rose Uchoa Rangel

**Affiliations:** 1 MDs, Department of Medicine, Universidade Federal de Sergipe (UFS), Aracaju, SE, Brazil.; 2 PhD, Associate Professor, Department of Medicine, Universidade Federal de Sergipe (UFS), Aracaju, SE, Brazil.; 3 PhD, Associate Professor, Department of Medicine, Universidade Federal de Sergipe (UFS), Aracaju, SE, Brazil. (In memoriam).

**Keywords:** Mesoblastic nephroma, Kidney, Preterm birth

## Abstract

A male child born at 27 weeks, weighting 1305 g and presenting with a right-sided abdominal tumor. Computed tomography scan demonstrated the presence of a solid mass compressing the right kidney. Puncture biopsy revealed congenital mesoblastic nephroma. The patient underwent total right nephroureterectomy, and died on the second day after surgery.

## Abstract (pt)

Criança do gênero masculino, nascida com 27 semanas e 1.305 g de peso, apresentando tumor abdominal à direita. Tomografia computadorizada revelou formação sólida comprometendo o rim direito. Foi realizada biópsia por punção, cujo diagnóstico foi nefroma mesoblástico congênito. O paciente foi submetido a nefroureterectomia total direita, falecendo no segundo dia de pós-operatório.

## INTRODUCTION

Congenital mesoblastic nephroma is rare renal neoplasia that was initially described by Bolande in 1967^(1,2)^. In spite of its low prevalence, congenital mesoblastic nephroma represents the most common renal neoplasm in neonates^(1,3)^. Such a tumor type is generally diagnosed at birth, or even between three and six months of age, is rarely found during childhood and exceptionally in the adulthood^(1,4)^. The prognosis is generally good, particularly in cases where total surgical resection (radical nephrectomy or nephroureterectomy) is performed^(3,4)^.

The present article reports a case of a mixed subtype of congenital mesoblastic nephroma with poor evolution, probably resulting from prematurity and postoperative septic shock.

## CASE REPORT

**Figure 1:**
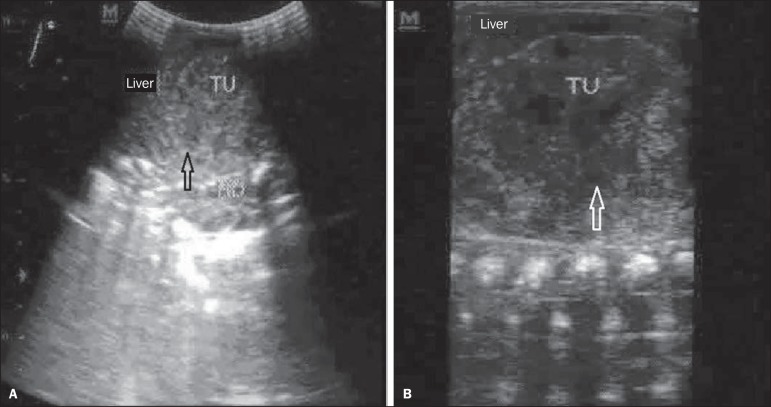
Total abdominal ultrasonography. **A:** Presence of heterogeneous tumor (TU) measuring approximately 5 × 4.7 × 4 cm located between the liver and the right renal locus (arrow). **B:** Presence of heterogeneous lesion, with hypoechoic areas predominantly located in the central region (arrow).

**Figure 2:**
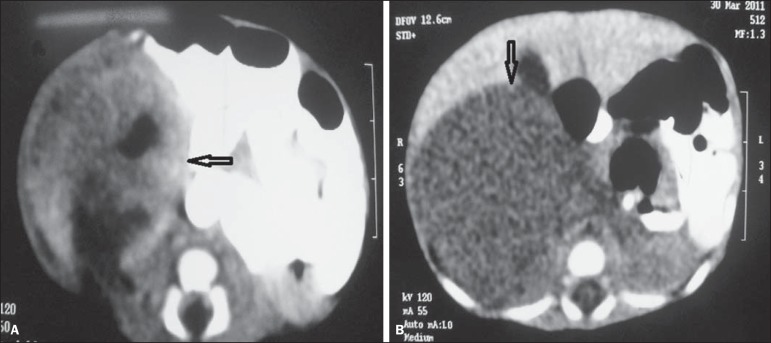
Abdominal computed tomography. **A:** Transverse section demonstrating the presence of a hypodense, solid mass measuring approximately 4 cm in diameter in the right renal locus (arrow). **B:** Transverse section demonstrating the presence of heterogeneous post-contrast enhancement in the region of the right kidney (arrow).

A male child born at the 27th gestational week, weighting 1305 g and with 36 cm in height. At physical examination, the child presented with globose abdomen, with a tumor at right, besides respiratory failure, and the patient was referred to the neonatal intensive care unit. Abdominal ultrasonography (US) demonstrated a solid tumor measuring 5.0 × 4.7 × 4.0 cm between the renal and hepatic regions (Figure 1). Abdominal computed tomography (CT) revealed the liver with a normal attenuation coefficient and presence of a solid mass with heterogeneous post-contrast enhancement compromising the right kidney, measuring about 4 cm in its largest diameter (Figure 2). Puncture biopsy was performed for pathological anatomy and immunohistochemical analyses. Histopathological analysis demonstrated spindle cell tumor with high mitotic index (10 mitoses/10 CGA). On the other hand, the immunohistochemical analysis revealed positive Ki-67 (10% of cells), CD99 and smooth muscle actin. The conclusion was classical/cell variants (mixed subtype) of congenital mesoblastic nephroma. Because of the histological type of the tumor, the neonate was submitted to total right nephroureterectomy. A bulky tumor occupying the whole renal locus, attached to the liver was found during the surgical procedure. The diaphragm was not involved. During the procedure, there was rupture of the tumor capsule, and the case evolved to the patient's death on the second postoperative day due to septic shock.

## DISCUSSION

Congenital mesoblastic nephromas (also known as Bolande's tumor) are most commonly found in male individuals, at a ratio of 2:1^(5)^. Such tumors probably originate from nephrogenic mesenchymal proliferation and generally present as an incidental finding of asymptomatic abdominal mass in neonates^(1,3)^. In some cases, tumor rupture may occur, leading to hemoperitoneum^(1,6)^. In some cases, the diagnosis may be made early in the prenatal period either by means of amniocentesis or US^(5,6)^. Besides tumor mass, sonographic findings include presence of polyhydramnios, sometimes in association with non-immune hydrops, which is generally fatal^(3)^.

Imaging methods play an important role in the post-natal diagnosis of mesoblastic nephromas^(7)^. Such methods can preoperatively assess the local invasion by the tumor, besides allowing to follow-up the patients for possible local recidivation^(8)^. Usually, US demonstrates a homogeneous, solid, well defined lesion with a gross appearance^(7,9)^. In some cases, typical small-sized mesoblastic nephromas may show a concentric pattern, alternating between hypo- and hyper-echoic rings^(10)^. The use of Doppler US allow for detecting intratumoral hypervascularization^(6)^.

CT allows for visualization of a solid renal mass with variable attenuation coefficient and possible necrotic and hemorrhagic areas, giving the lesion a heterogeneous appearance, which could indicate a worse prognosis^(11)^. The presence of small cystic areas is rarely observed, particularly in cases of typical mesoblastic nephromas^(7,9,10)^. Magnetic resonance imaging (MRI) is useful to identify invasion of local structures by the tumor. At MRI, mesoblastic nephromas present isosignal in relation to the renal cortex and to the skeletal muscle at T1-weighted images, and hypersignal in relation to those structures at T2-weighted images^(12)^. Excretory urography also is useful in the diagnosis of congenital mesoblastic nephromas, allowing for detecting distortion of the pyelocaliceal system caused by the presence of the renal mass^(10)^.

The differential diagnosis of congenital mesoblastic nephromas should be made with other renal neoplasias such as metanephric adenomas, renal clear cell carcinomas and Wilms' tumor^(8)^. Wilms' tumor and congenital mesoblastic nephromas are radiologically indistinguishable, so the differential diagnosis between such conditions is only possible by means of histopathology^(8)^.

Generally, mesoblastic nephromas are unilateral tumors, although in some cases they may be found bilaterally^(1)^. Macroscopically, the tumor is generally whitish and solid, with weight and volume ranging from 0.01 kg to 2 kg, and 150 to 200 cm^3^, respectively^(3,4)^. Two presentations of this tumor can be observed at the microscope, namely the classical and cellular patterns. At immunohistochemical analysis, congenital mesoblastic nephromas are positive for vimentin and smooth muscle actin, and negative for desmin, CD34, epithelial membrane antigen and cytokeratin^(1)^.

Usually, the treatment of this type of tumor is surgical, either with nephrectomy or total nephrouretectomy which, besides reducing the possibility of recurrence, will serve as treatment for the hypertension secondary to hyperreninemia^(3,6)^. With resection, the prognosis will be generally good, particularly in cases of classical histological subtype^(5)^.

Factors determining a poor prognosis are related to age, presence of positive surgical margins, and to a mixed histological type^(5)^.

In the present report, the case of a male, preterm neonate with a large, solid renal tumor at right is described. Imaging studies demonstrated the tumor with heterogeneous contrast enhancement. Histopathological analysis in association with immunohistochemical panel suggested the presence of a mixed subtype of congenital mesoblastic nephromas. The patient was submitted to total nephrouretectomy. Basically, except for the prematurity, all the other characteristics of the present case are in agreement with the literature.

The authors conclude that mesoblastic nephromas represents a rare renal neoplasia, and the knowledge on its clinical, radiological and histopathological characteristics is important to facilitate the differential diagnosis with other most common pediatric renal tumors.
